# Protective Effect of Edaravone on Glutamate-Induced Neurotoxicity in Spiral Ganglion Neurons

**DOI:** 10.1155/2016/4034218

**Published:** 2016-11-10

**Authors:** Xiaohui Bai, Chi Zhang, Aiping Chen, Wenwen Liu, Jianfeng Li, Qian Sun, Haibo Wang

**Affiliations:** ^1^Department of Otolaryngology-Head and Neck Surgery, Shandong Provincial Hospital Affiliated to Shandong University, Jinan 250021, China; ^2^Shandong Provincial Key Laboratory of Otology, Jinan 250022, China; ^3^Human Genetics Department, Emory University, Atlanta, GA, USA

## Abstract

Glutamate is an important excitatory neurotransmitter in mammalian brains, but excessive amount of glutamate can cause “excitotoxicity” and lead to neuronal death. As bipolar neurons, spiral ganglion neurons (SGNs) function as a “bridge” in transmitting auditory information from the ear to the brain and can be damaged by excessive glutamate which results in sensorineural hearing loss. In this study, edaravone, a free radical scavenger, elicited both preventative and therapeutic effects on SGNs against glutamate-induced cell damage that was tested by MTT assay and trypan blue staining. Ho.33342 and PI double staining revealed that apoptosis as well as necrosis took place during glutamate treatment, and apoptosis was the main type of cell death. Oxidative stress played an important role in glutamate-induced cell damage but pretreatment with edaravone alleviated cell death. Results of western blot demonstrated that mechanisms underlying the toxicity of glutamate and the protection of edaravone were related to the PI3K pathway and Bcl-2 protein family.

## 1. Introduction

Hearing loss is a very common sensory disorder which, to a great extent, influences the quality of patients' life. Sensorineural hearing loss is often associated with the impairment of spiral ganglion neurons (SGNs). SGNs are bipolar neurons that transmit auditory information from the ear to the brain. They are indispensable for the preservation of normal hearing and their survival depends mainly on genetic and environmental interactions [1]. Many disturbances, such as noise exposure, ototoxic medication, and genetic factors, can lead to the loss of SGNs irreversibly and therefore result in sensorineural hearing loss.

It is widely accepted that glutamate is an important excitatory neurotransmitter in mammalian brains, but excessive amount of glutamate can cause “excitotoxicity” and lead to neuronal death in some injuries and diseases, such as cerebral ischemia, traumatic brain disorder, HIV, and neurodegenerative disorders [2, 3]. Treatment with excessive glutamate in rats was found to result in high-frequency hearing loss. And there was a dramatic and selective reduction of neurons in the basal, high-frequency-related portion of the spiral ganglion, but no loss of hair cells was discovered [4]. Traumatic sound exposure, aminoglycoside antibiotics, cochlea ischemia, or traumatic stress leads to an excessive release of glutamate from inner hair cells into the synaptic cleft [5]. Glutamate excitotoxicity causes neuronal cell death primarily through the excessive activation of glutamate receptors which triggers massive Ca^2+^ influx into neurons [6]. Ca^2+^-loaded mitochondria generate reactive oxygen species (ROS), which comprises superoxide and nitric oxide [7, 8]. And large amount of ROS leads to cell death eventually.

Edaravone (MCI-186, 3-methyl-1-phenyl-2-pyrazolin-5-one) is a potent free radical scavenger which has already been used in the clinical treatment of ischemia impairments, such as acute cerebral infarction, acute myocardial infarction, and rheumatoid arthritis [9–11]. It can interact with both peroxyl and hydroxyl radicals to form oxidized compounds and thus attenuate ischemic damage [12]. Many studies have revealed that free radical scavengers are also very useful in the treatment of otology disorders, such as inner ear barotrauma, aminoglycoside-induced ototoxicity, and cisplatin-induced ototoxicity [13–15].

So far, few concerns have been focused on the protective effect of edaravone on spiral ganglion neurons against toxicity of glutamate. In this study, we aim to demonstrate whether edaravone, the free radical scavenger, can protect SGNs from glutamate-induced cytotoxicity and the possible underlying mechanism.

## 2. Materials and Methods

### 2.1. Materials

Dulbecco's Modified Eagle's Medium (DMEM) with high-glucose and fetal bovine serum were purchased from GIBCO (USA). Anti-NSE antibody was obtained from Abcam (USA); other antibodies including anti-Bcl-2, anti-Bax, anti-AKT, anti-p-AKT, and anti-*β*-actin were purchased from Santa Cruz Biotechnology (USA). Glutathione (GSH), superoxide dismutase (SOD), and malonaldehyde (MDA) assay kits (A006-1, A001-3, and A003-1, resp.) were all purchased from Nanjing Jiancheng Bioengineering Institute (China). BCA protein assay kit was a product from Shenergy Biocolor Bioscience & Technology Company (China). Glutamate (Glu), edaravone (Ed), 3-(4,5-dimethylthiazol-2-yl)-2,5-diphenyltetrazolium bromide (MTT), trypan blue solution, and other agents were obtained from Sigma (USA).

### 2.2. Primary Cultures of Rat SGNs

As our previous protocol [1], the SGNs cells were isolated from rats at age less than 5 postnatal days. After anesthesia with pentobarbital sodium, the rats were decapitated at the base of the foramen magnum, the epidermis was removed, cranium was opened along the sagittal suture, and the brain halves were removed. The following steps were carried out under the microscope and in phosphate buffered solution (PBS). The bulla of the temporal bone was opened, and then the capsule of the inner ear, the stria vascularis, and the organ of corti were removed. Rosenthal's canal was isolated and placed into Ca^2+^–Mg^2+^-free Hank's balanced salt solution containing 0.125% trypsinase. After digesting for 15 min at 37°C, the tissue was eluted with the plating medium DMEM supplemented with 10% fetal bovine serum. Then the cells were collected by centrifugation at 1000 rpm for 8 min, resuspended, and plated in poly-L-lysine-coated 24-well culture plates at a density of 1.0 × 10^5^ cells/mL at 37°C in a humid atmosphere of 5% CO_2_.

Next, primary SGNs were identified by immunocytochemistry staining. SGNs (1.0 × 10^5^/mL) were inoculated in 24-well plate, rinsed three times with PBS, and then stained with primary anti-NSE antibody (1 : 400) and secondary goat-anti-rabbit Cy3 antibody.

### 2.3. Drug Treatment

SGNs (1.0 × 10^5^/mL) subcultured in 96-well or 24-well plate were treated with 2 mM glutamate for 10 minutes. Then the medium was replaced by normal DMEM. Different concentrations of edaravone were added to the medium either 20 min before or 2 h, 6 h, and 12 h after glutamate treatment. All the doses and time points were determined by preliminary experiments (data not shown).

### 2.4. Assessment of Cell Viability by MTT and Trypan Blue Staining

Cell viability was quantified by MTT assay and trypan blue staining. MTT (5 mg/mL, 20 *μ*L) was added to each well and incubated for 4 h at 37°C after the drug treatments as described above. The medium was removed and the cell pellet was dissolved in DMSO. Then, the optical density (OD) values were measured at 570 nm using an ELISA reader. All experiments were repeated three times. Cell relative viability was calculated according to the following formula:

(1) $$\begin{array}{c} Cell\;\;relative\;\;viability\;\;\left(\;\%\;\right)=\frac{{OD}_{experiment}}{{OD}_{control}}\times 100\%. \end{array}$$

OD_blank_ was used as zero.

In trypan blue staining, SGNs were stained with 0.4% trypan blue for 5 min after the drug treatments as described above. Pictures were taken by microscope and trypan blue positive and negative cells were counted afterwards. Cell survival rate was defined as the percentage of negative cells.

### 2.5. Detection of Apoptosis and Necrosis by Ho.33342 and Propidium Iodide (PI) Double Staining

SGNs were incubated with glutamate with or without edaravone (500 *μ*M). Control cells were without any treatment. Cells were washed twice by PBS, fixed with 95% alcohol for 10 min, and then stained by Ho.33342 (10 mg/mL) and PI (50 mg/mL) at 37°C for 30 min. Morphological changes were examined by fluorescence microscope under green light (515–560 nm) and ultraviolet (UV) light (340–380 nm), respectively. At least 500 cells were counted in 5 randomly selected fields per group. All treatments were repeated three times.

### 2.6. Detection of GSH Content, SOD Activity, and MDA Level by Spectrophotometer

SGNs were incubated with 2 mM glutamate for 10 min with or without the pretreatment of 500 *μ*M edaravone 2 h ahead. Control cells were without any treatment. Then cells were washed twice with ice-cold PBS, sonicated, and harvested for the following assays. Intracellular GSH content, SOD activity, and MDA level in all groups were measured by commercial assay kits according to the manufacturer's instructions. OD values at optimal wavelengths were measured using spectrophotometer and the relative levels comparing with control cells were calculated. All experiments were repeated three times.

### 2.7. Protein Extraction and Western Blot Analysis

After SGNs were treated by 2 mM glutamate with or without pretreatment of 500 *μ*M edaravone, the proteins were collected and the expressions of AKT, p-AKT, Bax, and Bcl-2 genes were examined by western blot after 24 hours' normal culture. Briefly, total protein was extracted from SGNs using lysis buffer (containing 50 mM Tris-Cl, pH 8.0, 150 mM NaCl, 0.1% SDS, 1% NP-40, and 100 mg/mL PMSF). The protein concertation of each sample was measured by BCA protein assay kit. Total protein 40 mg of each sample was loaded in 10% SDS-PAGE gels and electrically transferred onto polyvinylidene difluoride membranes. After that, the membranes were blocked in 5% nonfat dried milk/Tris-buffered Saline-Tween for 2 h at room temperature. Subsequently, the membranes were incubated with primary antibodies for 2 h at room temperature (anti-AKT: 1 : 500, anti-p-AKT: 1 : 400, anti-Bcl-2: 1 : 400, anti-Bax: 1 : 400, and anti-*β*-actin: 1 : 2000). Following three washes with TBST, the blots were incubated with the secondary goat-anti-mouse or goat-anti-rabbit IgG antibody (1 : 2000) at room temperature for 2 h. Finally, the immunoblots were detected by an ECL kit and visualized after exposure to X-ray film. Densitometer was used to quantitate the immunoreactive bands. The ratios of AKT, p-AKT, Bcl-2, and Bax to*β*-actin were then determined.

### 2.8. Statistical Analysis

Data were presented as mean ± standard error on the mean (SEM). Statistical calculations were performed using SPSS19.0 software. One-way analysis of variance (ANOVA) was applied to analyze data. *p* < 0.05 was considered statistically significant.

## 3. Results

### 3.1. SGNs Were Identified by Anti-NSE Antibody

**Figure 1:**
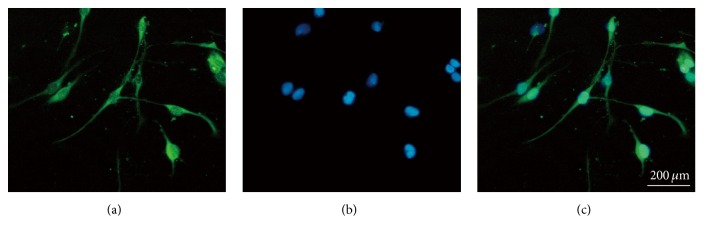
Identification of SGNs by anti-NSE antibody. (a) Corresponding NSE-Cy3 field to identify NSE positive cells. (b) Corresponding DAPI field, showing all nucleoli in the field. (c) (a), and (b) merged.

Figure 1 showed all nuclei stained by DAPI presented blue florescence; meanwhile, the cells stained by NSE presented green fluorescence. Merging of the stained images showed the cells with blue-green fluorescence, which were identified as SGNs.

### 3.2. Edaravone Performed Both Preventative and Therapeutic Effects against Toxicity of Glutamate

**Figure 2:**
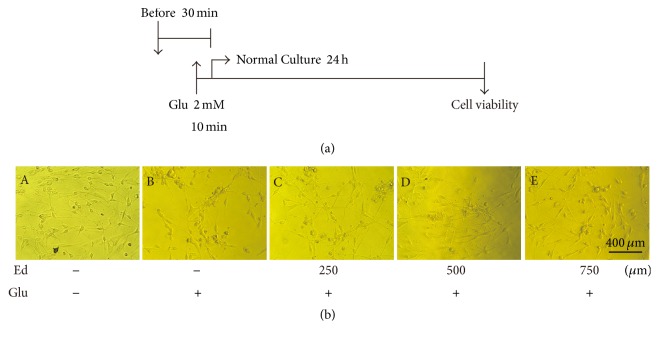
Preventative effect of edaravone on toxicity of glutamate in cultures of SGNs. (a) Illustration of drug treatment. SGNs were treated with edaravone first and 2 mM glutamate 20 min later. Then the medium was changed to normal medium 10 min after that. Cell viability was observed after normal culture for 24 h. (b) Pretreatment with edaravone mitigated cell death and morphological changes caused by glutamate. (A) The normal-cultured control cells. (B, C, D, and E) Morphological changes in SGNs treated with glutamate and different concentrations of edaravone (0, 250, 500, and 750 *μ*M) were observed, respectively.

To determine whether edaravone has preventative effect on glutamate-induced cell damage, 4 experimental groups of SGNs were arranged. One group was treated with 2 mM glutamate alone for 10 min while the other three groups were pretreated with edaravone at different concentrations (250 *μ*M, 500 *μ*M, and 750 *μ*M) for 20 min before glutamate treatment. Then the medium was changed to normal medium (Figure 2(a)). For the control group, SGNs were cultured in normal medium without any treatments. SGNs treated with glutamate appeared obvious morphological changes compared with control group when observed under phase contrast microscope. In addition, with glutamate treatment, the number of cells was significantly decreased, many cells were dying, lost the fusiform shape, and became round or elliptical, and large quantities of dead cells were also observed. Pretreatment of edaravone reversed these changes resulting from glutamate treatment, and at the dose of 500 *μ*M and 750 *μ*M, SGNs showed favorable growth without distinct cell death (Figure 2(b)).

**Figure 3:**
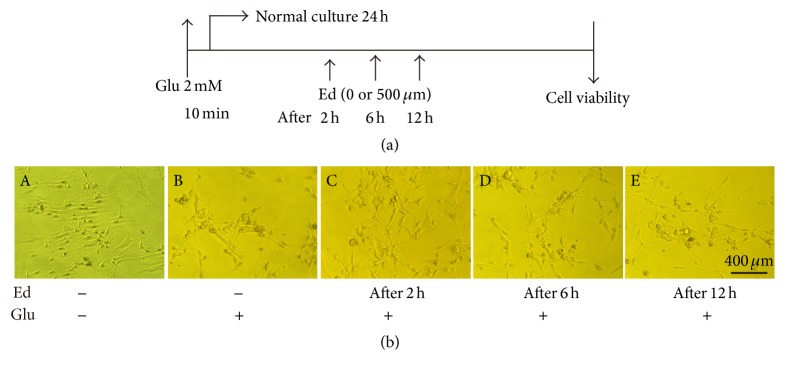
Therapeutic effect of edaravone on toxicity of glutamate in cultures of SGNs. (a) Illustration of drug treatment. SGNs were treated with 2 mM glutamate for 10 min; then the medium was changed to normal medium. 500 *μ*M edaravone was added to the medium at different time points (2 h, 6 h, and 12 h later), respectively. Cell viability was observed after normal culture for 24 h. (b) Treatment with edaravone reduced cell death and morphological changes of SGNs caused by glutamate. (A) The control cells. (B, C, D, and E) Morphological changes in SGNs treated with glutamate and 500 *μ*M edaravone added at different time point.

Based on the result, we considered the 500 *μ*M concentration of edaravone to be desirable and chose it to do the following experiments. In order to make sure that edaravone also had therapeutic effect on glutamate-induced toxicity, all the groups of SGNs were treated with glutamate for 10 min first, and then the medium was changed to normal medium. 500 *μ*M edaravone was added to the medium 2 h, 6 h, or 12 h after glutamate treatment (Figure 3(a)). Cell death decreased with the treatment of edaravone. 2 h after glutamate treatment or even earlier time point was the most suitable time point for administering edaravone to reach the maximal protection effect among these groups. At later time points, the cell death could not be reduced effectively (Figure 3(b)). In short, these results showed that edaravone performed both preventative and therapeutic effects on the glutamate-induced toxicity in SGNs.

### 3.3. Edaravone Alleviated the Decrease of Cell Viability Caused by Glutamate

**Figure 4:**
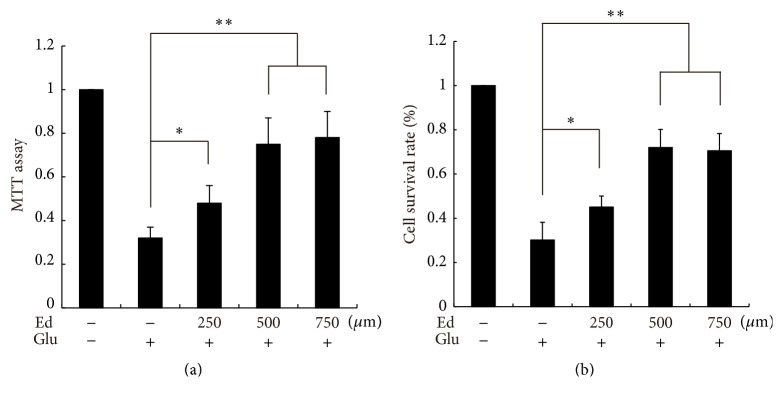
Pretreatment of edaravone reduced the toxicity of glutamate towards SGNs. Cell viability was detected by MTT assay (a) and trypan blue staining (b). Three groups were pretreated with 250 *μ*M, 500 *μ*M, and 750 *μ*M edaravone, respectively. ^*∗*^ *p* < 0.05, ^*∗∗*^ *p* < 0.01, versus glutamate-treated group. The data shown here was the mean ± SEM of three separate experiments.

**Figure 5:**
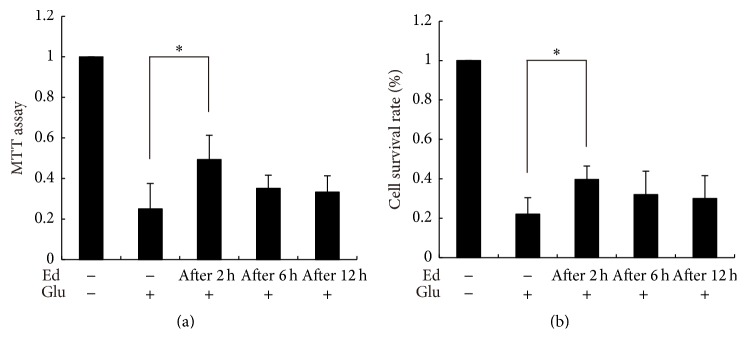
Treatment of edaravone buffered the toxicity of glutamate towards SGNs. Cell viability was detected by MTT assay (a) and trypan blue staining (b). Three groups were treated with 500 *μ*M edaravone 2 h, 6 h, and 12 h after administration of glutamate, respectively. ^*∗*^ *p* < 0.05, versus glutamate-treated group. The data shown here was the mean ± SEM of three separate experiments.

In order to estimate the protective effect of edaravone, MTT assay and trypan blue staining were performed to measure cell viability. The cell viability of control group was considered as 100%.  Figure 4 showed that one group of SGNs was treated with glutamate alone, while the other three groups were pretreated with edaravone at different concentrations (250 *μ*M, 500 *μ*M, and 750 *μ*M) for 20 min before glutamate treatment. The cell viabilities of MTT test were 32%, 48%, 75%, and 78% for each group, respectively (Figure 4(a)), and those of trypan blue staining were 30%, 45%, 72%, and 70% (Figure 4(b)). These results revealed that pretreatment with edaravone increased the cell viability of SGNs and the protective effect was presented in a dose-dependent manner. The protection reached the peak at the concentration of 500 *μ*M and no obvious benefits were observed by further elevating the concentration.  Figure 5 showed that one group of SGNs was treated with glutamate alone, while the other three were treated with 500 *μ*M edaravone 2 h, 6 h, or 12 h after glutamate treatment, respectively. The cell viabilities of MTT test were 25%, 49%, 35%, and 33%, respectively (Figure 5(a)), and those of trypan blue staining were 22%, 40%, 32%, and 30% (Figure 5(b)). Treatment of edaravone at the time point of 2 h after glutamate achieved satisfying protection for SGNs against glutamate-induced cytotoxicity. But there was no significant improvement in cell viability when treated at later time points.

### 3.4. Edaravone Reduced Apoptosis and Necrosis Caused by Glutamate

**Figure 6:**
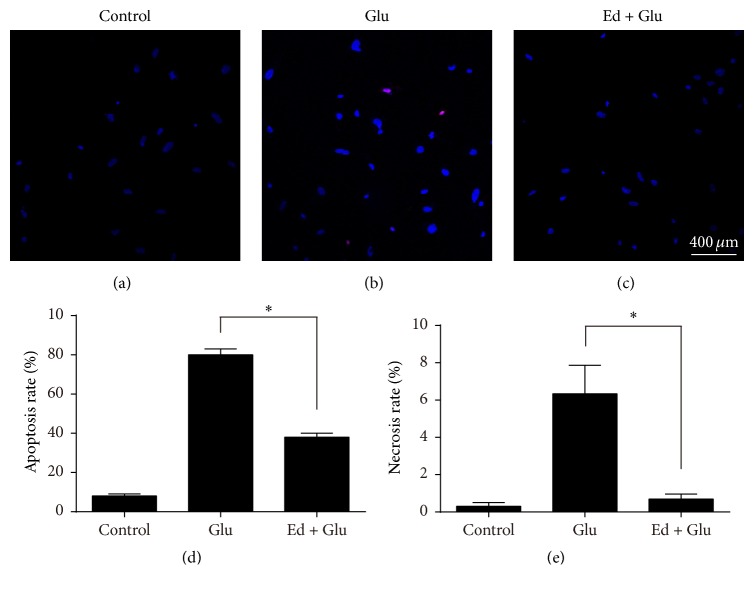
Cytoprotection of edaravone on glutamate-induced apoptosis and necrosis in SGNs. (a) Untreated SGNs appeared blue intact nuclei. (b) SGNs were treated with glutamate for 10 min and then cultured for 24 h. Nuclei of apoptotic SGNs were obvious and stained brilliant-blue by Ho.33342. Nuclei of necrotic cells were labeled red by PI. (c) SGNs were pretreated with 500 *μ*M edaravone 20 min before glutamate. (d and e) Apoptosis and necrosis rates of SGNs. ^*∗*^ *p* < 0.05, versus glutamate-treated group.

Apoptosis and necrosis were detected using Ho.33342 and PI. Nuclei of apoptotic cells would be stained with brilliant-blue fluorescence by Ho.33342, while nuclei of necrotic cells would be stained with red fluorescence by PI. After treatment with 2 mM glutamate for 10 min, the cells were changed to normal medium and culture for additional 24 h. Then SGNs were fixed and stained with Ho.33342 and PI. Nuclei which were dyed brilliant-blue or red demonstrated the occurrence of apoptosis or necrosis. The percentages of necrotic cells and apoptotic cells with glutamate treatment were higher than those of the control group without any treatment. On the contrary, SGNs pretreated with edaravone compared to control group showed no obvious apoptosis and necrosis (Figure 6). So we came to the conclusion that glutamate could induce apoptosis and necrosis of SGNs, but edaravone could effectively alleviate cell death.

### 3.5. Edaravone Reversed Decrease of SOD Activity, MDA Elevation, and GSH Reduction Caused by Glutamate

**Figure 7:**
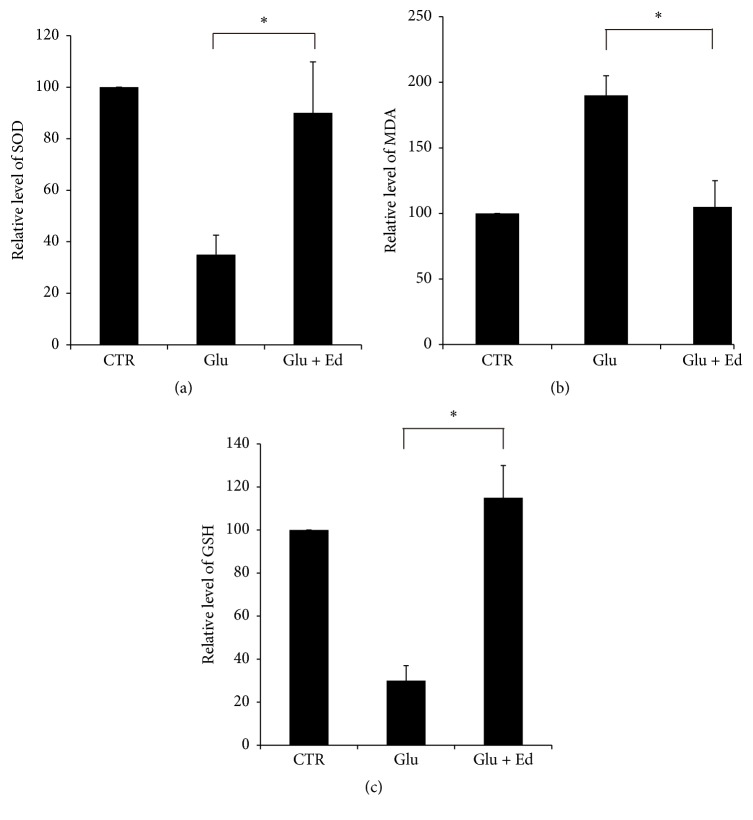
Changes of SOD activity, MDA level, and GSH content in different groups. Compared with the control group, glutamate decreased activity of SOD, content of SOD, and elevated level of MDA markedly. Edaravone protected SGNs by reversing these changes. ^*∗*^ *p* < 0.05. The data shown here was the mean ± SEM of three separate experiments.

By taking control as 100%, treatment of SGNs with 2 mM glutamate decreases activity of SOD to 35% and level of GSH to 30% and increased content of MDA to 190%. Pretreatment of edaravone (500 *μ*M) reversed these changes to approximately normal levels, with activity of SOD to 90%, level of GSH to 115%, and content of MDA to 105% (Figure 7). These changes were all statistically significant (^*∗*^ *p* < 0.05).

### 3.6. Edaravone Protected SGNs from Glutamate-Induced Apoptosis through PI3K/Akt Pathway

**Figure 8:**
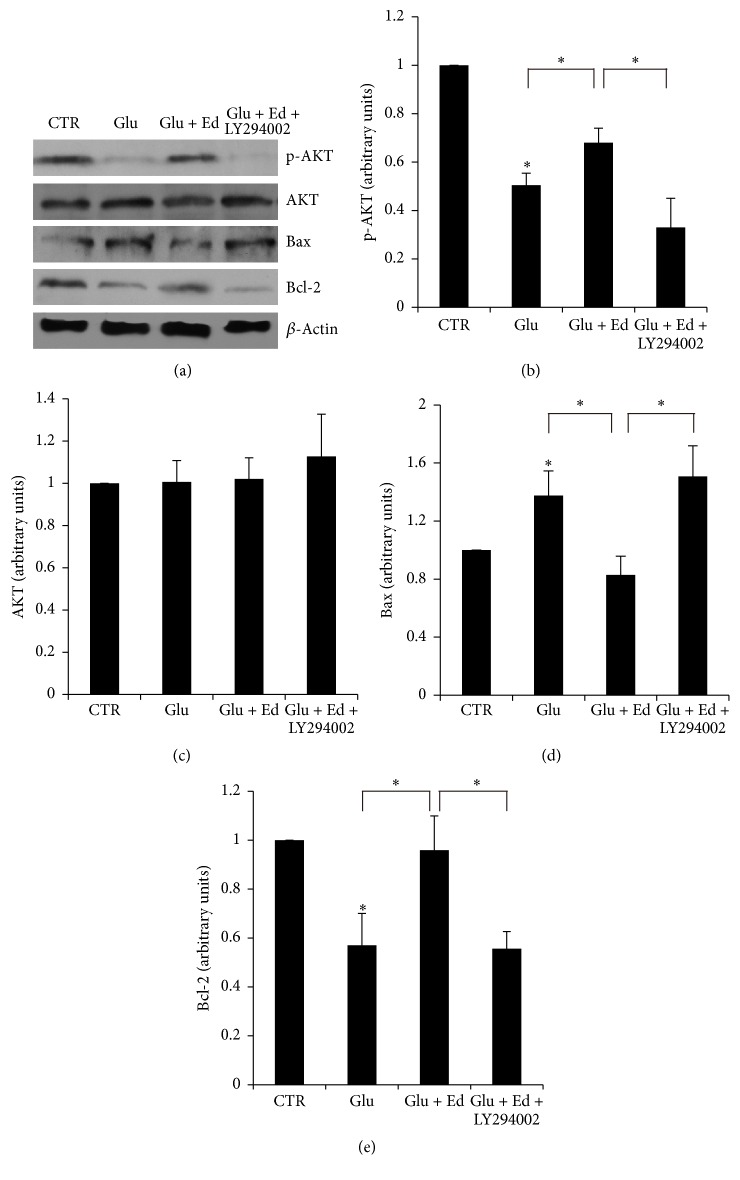
Detection of expression of p-AKT, AKT, Bax, and Bcl-2 by western blot. (a) Representative blots. (b, c, d, and e) Quantification of immunoreactive bands by densitometer. Treatment with glutamate resulted in the reduction of p-AKT, Bcl-2, and elevation of Bax. Pretreatment with 500 *μ*M edaravone protected SGNs by reversing these changes. Meanwhile, LY294002 eliminated the protection effect of edaravone. Statistical analyses were carried out among different groups. ^*∗*^ *p* < 0.05.

As shown in Figure 8, western blot analysis was performed in order to demonstrate the mechanism of edaravone's antiapoptotic effect. Representative blots showed the amount of p-AKT, AKT, Bcl-2, and Bax in SGNs (Figure 8(a)) and densitometer was used to quantitate the immunoreactive bands (Figures 8(b), 8(c), 8(d), and 8(e)). Treatment of SGNs with glutamate reduced AKT phosphorylation significantly. Moreover, the expression of antiapoptotic protein Bcl-2 was increased and the apoptotic protein Bax was decreased. Pretreatment of SGNs with 500 *μ*M edaravone reversed these changes. The protection of edaravone could be blocked by the PI3K inhibitor, LY294002. Therefore, the protective effect of edaravone on SGNs against glutamate-induced apoptosis was associated with PI3K/Akt pathway and Bcl-2 protein family.

## 4. Discussion

Ischemic brain injury can cause glutamate accumulation, and then postsynaptic glutamate receptors are overstimulated and intracellular Ca^2+^ overload occurs. This succession leads to the generation of free radicals and finally cell death. Since administration of glutamate in rats led to high-frequency hearing loss [4] and glutamate might be an important neurotransmitter in cochlea [16], in some cases glutamate accumulation may be a pathogenic mechanism of some otology disorders. Thus, whether free radical scavengers, such as edaravone, can protect SGNs from glutamate-induced cell damage is a meaningful question to be solved. Edaravone was the first free radical scavenger that has provided clinical evidence for therapeutic effects on ischemic stroke and it has been used clinically since 2001 [17]. Edaravone has been previously reported to protect several organs, such as the brain [18], kidney [19], liver [20], and retina [21] from free radical-induced damage. It has also been proven to be useful in otology disorders. Streptomycin-induced vestibulotoxicity in guinea pig could be attenuated by edaravone [22]. And edaravone could protect cochlea from acoustic trauma induced by reactive oxygen species [23]. In this study, we discovered that edaravone could protect spiral ganglion neurons from glutamate-induced cell damage, and the underlying mechanism was related to PI3K pathway and proteins of Bcl-2 family.

SGNs were identified first by NSE antibody through immunofluorescence analysis. Treatment of SGNs with glutamate induced obvious morphological changes and large percentage of cell death, and these changes were reversed by edaravone administration both before and after glutamate treatment (Figures 2 and 3). This phenomenon gave us the clue that edaravone could elicit both preventative and therapeutic effects against glutamate-induced cell damage.

In order to further clarify the protective effect of edaravone, MTT assay and trypan blue staining were performed to examine the cell viability of glutamate-treated SGNs with or without combining with edaravone treatment. In the living cells, the mitochondria can change MTT into blue crystal and trypan blue cannot pass the intact cell membrane, while the opposite happens in the damaged cells. The results showed that treatment with glutamate resulted in severe reduction of cell viability indicating massive cell death. Pretreatment of edaravone significantly decreased the glutamate-induced toxicity and elevated the cell viability markedly in a dose-dependent manner. The protection reached the peak at the concentration of 500 *μ*M and no obvious improvement was observed at higher concentrations (Figure 4(a)). Treatment with edaravone 2 hours after glutamate also reduced cell death significantly but no obvious differences were observed in later time points. These results demonstrated that treatment with edaravone before or after glutamate can decrease glutamate-induced cell death significantly in SGNs. The preventative effect of edaravone at 500 *μ*M was quite satisfying and this management was employed in the following experiments.

Apoptosis and necrosis are two typical forms of cell death. Ankarcrona et al. discovered that glutamate-induced neuronal death was a succession of necrosis or apoptosis depending on mitochondrial function [3]. Glutamate also induced apoptosis in spiral ganglion explants and the apoptosis could be prevented by a caspase-3 inhibitor [5]. In this study, Ho.33342 and PI staining revealed that both apoptosis and necrosis took place after administrating glutamate. Furthermore, apoptotic cells which were stained with brilliant blue color accounted for the majority of cell death indicating that apoptosis was the predominant form of cell damage induced by glutamate in SGNs. Pretreatment with edaravone reduced the glutamate-induced apoptosis and necrosis.

Next, we investigated the possible mechanism underlying glutamate's excitotoxicity and edaravone's protection on SGNs. Oxidative stress is a common underlying process related to a variety of disorders, such as ischemia-reperfusion disorders, cardiovascular diseases, cancer, and diabetes mellitus. It is well known that GSH and SOD are critical components in fighting against oxidative stress. MDA is the product of lipid peroxidation which is initiated in the presence of hydroxyl radicals. These are all important indicators of oxidative stress. So we then measured the changes of SOD activity, MDA level, and GSH content in different experimental groups. The results showed that, after treatment of glutamate, SOD activity and GSH content were reduced, while MDA level was elevated significantly, which meant oxidative stress played an important role in glutamate-induced cell damage. Meanwhile, pretreatment of edaravone reversed these changes to almost normal levels.

PI3K/Akt pathway is an important antiapoptotic pathway. Results of western blot showed that treatment of SGNs with glutamate inhibited the phosphorylation of Akt, when the level of total Akt remained constant. Bcl-2 and Bax were considered to be involved in the antiapoptotic effect and neural protection of edaravone [24, 25]. Treatment of SGNs with glutamate resulted in the elevation of the apoptotic protein Bax and the reduction of antiapoptotic protein Bcl-2. Pretreatment with edaravone eliminated all of these phenomena above. In addition, LY294002, the PI3K inhibitor, was used to block PI3K pathway and eventually erased the protection of edaravone. This result indicated that PI3K pathway and Bcl-2 protein family were related to the protection effect of edaravone in glutamate-induced cytotoxicity of SGNs.

Therapies against glutamate-induced cell damage have been discussed widely. Local application of glutamate receptor antagonists, such as caroverine, showed a therapeutic effect when applying 1 h after noise exposure but not 24 h afterwards [26]. Glutamate-induced apoptosis could be blocked selectively by a caspase-3 inhibitor in cultured spiral ganglion explants [5]. In this study, administration of edaravone before or after glutamate presented desirable protective effects in SGNs. It reduced apoptosis and necrosis significantly and reversed the changes of SOD, GSH, and MDA measurements. So, we believe edaravone or other free radical scavengers could be an option in the treatment of sensorineural hearing disorders related to glutamate accumulation.

## 5. Conclusion

In this study, we discovered that glutamate induces both apoptosis and necrosis in spiral ganglion neurons in vitro, but apoptosis is the main form of cell death. Edaravone is a potent free radical scavenger and can elicit salient protective effect against glutamate-induced cell damage in SGNs. The underlying mechanism is associated with the PI3K pathway and Bcl-2 protein family. The combination of free radical scavenger, such as edaravone, glutamate antagonist, and caspase-3 inhibitor, may be a desirable treatment of hearing disorders induced by an excessive release of glutamate.
